# Determination of virulence and fitness genes associated with the *pheU*, *pheV* and *selC* integration sites of LEE-negative food-borne Shiga toxin-producing *Escherichia coli* strains

**DOI:** 10.1186/s13099-018-0271-8

**Published:** 2018-10-08

**Authors:** Nadja Saile, Elisabeth Schuh, Torsten Semmler, Inga Eichhorn, Lothar H. Wieler, Andreas Bauwens, Herbert Schmidt

**Affiliations:** 1 Institute of Food Science and Biotechnology, University of Hohenheim, Garbenstr. 28, 70599 Stuttgart, Germany; 2 Department Biological Safety, National Reference Laboratory for Escherichia coli, Federal Institute for Risk Assessment (BfR), Diedersdorfer Weg 1, 12277 Berlin, Germany; 3 Robert Koch Institute, Nordufer 20, 13353 Berlin, Germany; 4 Institute of Microbiology and Epizootics, Freie Universität Berlin, Robert-von-Ostertag-Str. 7-13, 14163 Berlin, Germany; 5 Institute for Hygiene, University of Münster, Robert-Koch-Str. 41, 48149 Münster, Germany

**Keywords:** STEC, Food, *pheU*, *pheV*, *selC* integration site, Genomic island

## Abstract

**Background:**

In the current study, nine foodborne “Locus of Enterocyte Effacement” (LEE)-negative Shiga toxin-producing *Escherichia coli* (STEC) strains were selected for whole genome sequencing and analysis for yet unknown genetic elements within the already known LEE integration sites *selC*, *pheU* and *pheV*. Foreign DNA ranging in size from 3.4 to 57 kbp was detected and further analyzed. Five STEC strains contained an insertion of foreign DNA adjacent to the *selC* tRNA gene and five and seven strains contained foreign DNA adjacent to the *pheU* and *pheV* tRNA genes, respectively. We characterized the foreign DNA insertion associated with *selC* (STEC O91:H21 strain 17584/1), *pheU* (STEC O8:H4 strain RF1a and O55:Hnt strain K30) and *pheV* (STEC O91:H21 strain 17584/1 and O113:H21 strain TS18/08) as examples.

**Results:**

In total, 293 open reading frames partially encoding putative virulence factors such as TonB-dependent receptors, DNA helicases, a hemolysin activator protein precursor, antigen 43, anti-restriction protein KlcA, ShiA, and phosphoethanolamine transferases were detected. A virulence type IV toxin-antitoxin system was detected in three strains. Additionally, the *ato* system was found in one strain. In strain 17584/1 we were able to define a new genomic island which we designated GI*selC*_17584/1_. The island contained integrases and mobile elements in addition to genes for increased fitness and those playing a putative role in pathogenicity.

**Conclusion:**

The data presented highlight the important role of the three tRNAs *selC*, *pheU,* and *pheV* for the genomic flexibility of *E. coli*.

**Electronic supplementary material:**

The online version of this article (10.1186/s13099-018-0271-8) contains supplementary material, which is available to authorized users.

## Background

The genome sequence of the *Escherichia coli* type strain (U5/41T) was recently published and revealed a size of about 5 Mbp, containing 4762 protein-coding genes [1]. In comparison to the non-pathogenic *E. coli* strains with genome sizes of 4.5–5 Mbp, pathogenic *E. coli* frequently carry additional DNA and reach genome sizes up to 5.9 Mbp [2, 3]. This additional DNA is often located on plasmids, prophages or genomic islands, all of which might be acquired by horizontal gene transfer [4]. Genomic islands (GEI) are distinct DNA regions that are usually larger than 30 kbp, and their GC-content differs from the host genome. They are often flanked by insertion sequences or direct repeats. When additional pathogenicity-associated genes are present, they are also referred to as pathogenicity islands (PAIs). PAIs are found exclusively in pathogenic members of a species, contain multiple (cryptic) mobile genetic elements, have fitness or virulence factors and are often associated with tRNA loci which function as integration sites for additional DNA. Such PAI are summarized as “foreign DNA” in the current study [5, 6]. Comparative genomics studies of a group of Shiga toxin-producing *E. coli* (STEC), the so-called enterohemorrhagic *E. coli* (EHEC), were performed by Ogura and colleagues and revealed that EHEC possess more tRNA genes than other *E. coli* and *Shigella* strains. Additionally, they showed that tRNA genes are often the target of DNA insertions, often as insertion loci for two or three genetic elements at once [3, 7]. The best described PAI in EHEC which is inserted adjacent to a tRNA gene is the “Locus of Enterocyte Effacement” (LEE) with a basic size of about 35 kbp. The genes of this pathogenicity island are often close to the tRNA genes *selC*, *pheU* or *pheV* [8]. The LEE is an important virulence factor, and its gene products support the intimate binding of the bacterium to the host cell and the release of several effector proteins into the host cell cytoplasm [8–10]. Besides the LEE, researchers have also described other PAIs as being integrated into the *selC* locus. These include PAI-1 in the uropathogenic *E. coli* (UPEC) strain 536 and SPI-3 in *Salmonella enterica* as well as the 33 kbp locus of proteolysis activity in the STEC strain 4797/97 and the toxigenic invasion locus A in the enterotoxigenic *E. coli* (ETEC) strain H10407 [11–14]. Others have also found an attachment site of the *E. coli* retronphage ΦR73 integrated into *selC* [15]. For uropathogenic *E. coli* strains, the percentage of genomic islands is nearly 13% of the genome, demonstrating the importance of horizontally acquired DNA [16]. Strain CFT073, for example, was shown to harbour 13 genomic islands larger than 30 kbp. One of them is integrated in *selC* with a size of 68 kbp and is named intC-c4581 [16].

Both integration sites *pheU* and *pheV* have an identical sequence but differ in their gene surroundings. For *pheU* and *pheV*, different variants of the LEE locus with sizes between 36 and 111 kbp have been described [8].

One of these is the hybrid PAI I_CL3_, which contains the LEE core, parts of two different genomic islands detected in EHEC strain EDL933 (OI-48 and OI-122) and DNA homologous to *Yersinia pestis.* The PAI I_CL3_ is integrated in *pheV* of *Citrobacter rodentium* and in STEC [17, 18]. Another PAI originally found in *pheU* but more often detected in *pheV* was described as including an adhesin encoded by an *afa*-*8* gene cluster which was found in human and bovine *E. coli* isolates [19]. Uropathogenic *E. coli* (UPEC) and *Shigella* strains were also shown to harbour genomic islands at these integration sites. In UPEC J96, the PAI V_J96_ with 110 kbp is located adjacent to *pheU* and the PAI IV_J96_ with more than 170 kbp was detected at the *pheV* integration site [20]. *Shigella boydii* owns an iron transport-associated PAI of 21 kbp named SHI-3 in *pheU* and *Shigella flexneri* 2a a PAI of 46.6 kbp in *pheV* [21, 22].

STEC are important foodborne pathogens with more than 400 described serotypes and a high diversity of isolates from contaminated food [23].

Therefore, the aim of the study was to investigate whether LEE-negative foodborne STEC strains harbour foreign DNA at the LEE integration sites and whether this DNA may contribute to the fitness and pathogenicity of these strains. Nine strains with at least one occupied integration site *selC*, *pheU* or *pheV* were selected and subjected to whole genome sequencing and characterization of their foreign DNA.

## Methods

### Bacterial strains and culture conditions

The foodborne STEC strains TS18/08, LM27558_stx2_, RF1a, TS25/08, LM27564, LM14603/08, K30, TS21/08 and 17584/1 have been isolated from risk foods (Table 1) and were selected because they were LEE-negative and a former study had shown that at least one of the tRNA sites *selC*, *pheU* or *pheV* was occupied by additional DNA [24, 25]. The strains were cultured overnight in LB broth (1% (w/v) tryptone, 0.5% (w/v) yeast extract, 1% (w/v) NaCl, pH 7) at 37 °C with agitation at 180 rpm. DNA was isolated using a Qiagen Blood and Tissue Kit following the manufacturer’s instructions (Qiagen, Hilden, Germany). DNA concentration and purity were measured using a Nanodrop 2000 device (Thermo Fisher Scientific, Schwerte, Germany).

**Table 1:** General characteristics of LEE-negative foodborne STEC used for sequence analysis and the respective accession numbers

Strain^a^	Serotype^a^	Source^a^	Predicted occupied integration sites^b^	No. of contigs	N50 value (kbp)	Calculated genome size (Mbp)	Gen Bank Accession number
TS18/08	O113:H21	Minced meat	*pheV*	226	150	5.1	MPTX00000000
LM27558_stx2_	Orough:H43	Deer meat	*selC, pheU, pheV*	410	111	5.7	MPTY00000000
RF1a	O8:H6	beef	*selC, pheU*	233	173	5.2	MPTZ00000000
TS25/08	Ont:Hnt	Minced meat	*pheV*	419	79	5.2	MPUA00000000
LM27564	O113:Hnm	Deer meat	*pheU, pheV*	413	101	5.4	MPUB00000000
LM14603/08	O21:H21	Deer meat	*selC, pheU, pheV*	271	169	5.4	MPUC00000000
K30	O55:Hnt	Raw milk	*pheU*	181	224	5.0	MPUD00000000
TS21/08	O113:H21	Minced meat	*selC, pheV*	297	177	5.2	MPUE00000000
17584/1	O91:H21	Mettwurst	*selC, pheV*	341	140	5.0	MPUF00000000

*nt* not typeable, *nm* non motile

^a^Data from Slanec et al. [25]

^b^Data from Hauser et al. [24]

### Whole genome sequencing and sequence analysis

The concentration of the purified DNA was evaluated using the Qubit dsDNA HS Assay (Life Technologies, Darmstadt, Germany). MiSeq libraries containing 1 ng of DNA were prepared with Nextera XT chemistry (Illumina, San Diego, CA, USA) and were sequenced in a paired-end run (2 × 300 bp) on an Illumina MiSeq sequencer as recommended by the manufacturer with a minimum coverage of 90×. Raw data was de novo assembled using CLC Genomics Workbench (http://www.qiagenbioinformatics.com) resulting in assemblies with N50 values between 79 and 224 kbp consisting of between 181 and 419 contigs. The draft genomes were further analysed using Geneious software ver. 7.1, 9.1.8 and 10.0.7 (http://www.geneious.com). Annotations were carried out using the RASTk annotation tool within the PATRIC web resources [26–28]. For comparisons, the Blastn and Blastx algorithms were used (http://blast.ncbi.nlm.nih.gov). The genome sequences of the investigated strains have been deposited in the NCBI database under the accession numbers given in Table 1. For integration site detection and description, *selC*, *pheU* and *pheV* site-specific primers sequences, which were described before, were used for in silico analyses [24]. For strain LM14603/08 the *pheU* and *pheV* integration sites were confirmed for DNA insertion by PCR, as recently described [24].

## Results

In this study, nine LEE-negative foodborne STEC strains of different serotypes (Table 1) were investigated by whole genome sequencing to gain further insight into the identity of additional DNA at LEE integration sites. For each strain, at least one of the integration sites *selC*, *pheU* or *pheV* was occupied as previously analysed by PCR. Using the respective primers, strains without integration of additional DNA in those integration sites showed the expected sequences and amplicon sizes (*selC* locus 2173 bp, *pheU* locus 664 bp, *pheV* locus 1306 bp).

### General characteristics of LEE-negative food-borne STEC

Whole genome sequencing was performed with nine STEC strains and the number of contigs achieved range from 181 to 419 among the analysed strains. More detailed information is given in Table 1.

**Fig. 1:**
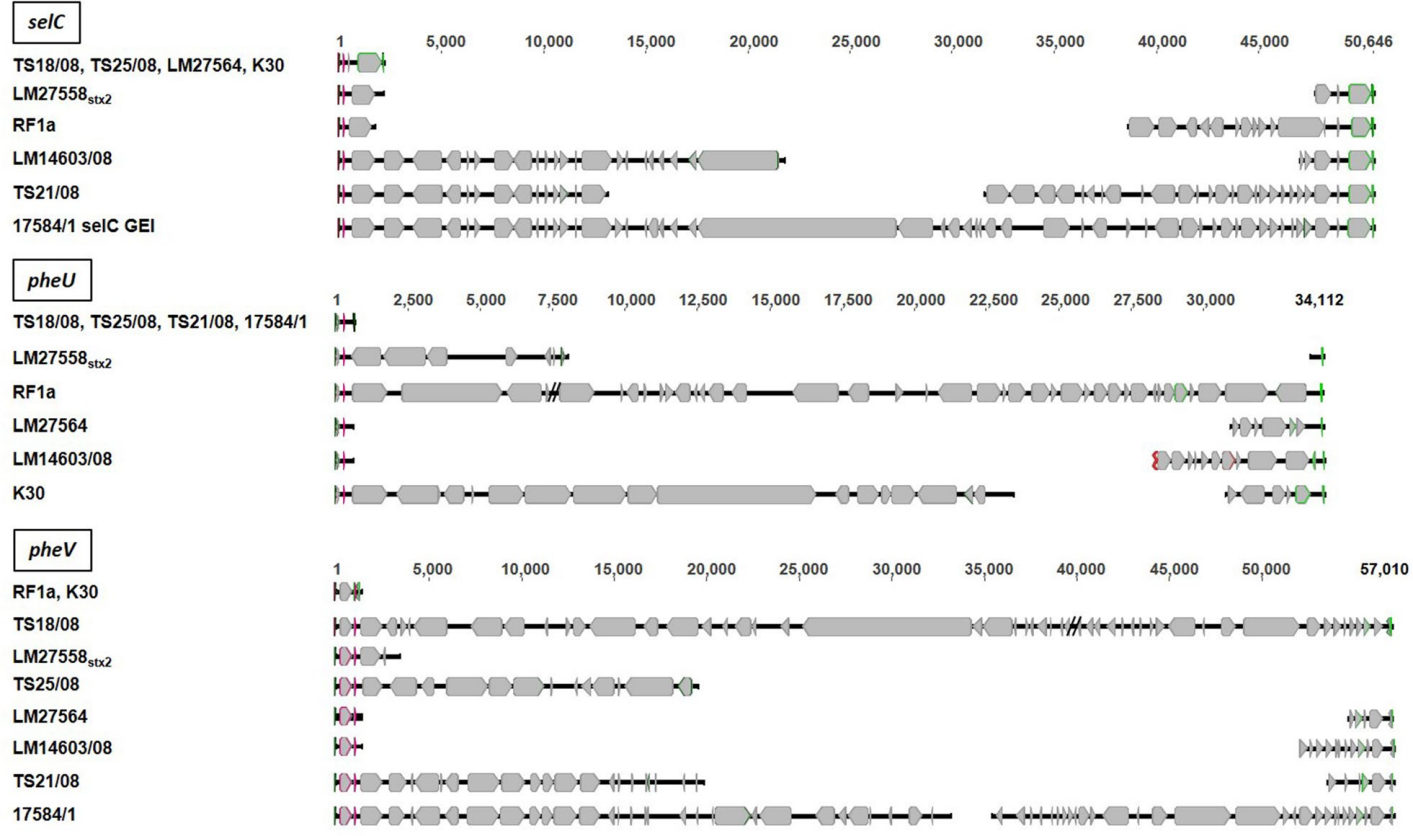
Schematic overview of integrated DNA adjacent to tRNA-genes *selC*, *pheU* and *pheV* in foodborne STEC strains. On the left side are the strain designations and integration site. On the right side are the top of each of the three groups’ nucleotide counts for the integrated DNA with schematic view of the respective contigs for each strain (black lines) including CDS (grey arrows). The first row of each group shows strains without additional DNA (in green forward and reverse primer for detection, in pink tRNA gene). The black double slash shows the gap between contigs

In agreement with the PCR results, five of the nine strains contained additional DNA in *selC*, five strains within *pheU* and seven within *pheV*. Strain LM14603/08 was found to harbour additional DNA in *pheU* and *pheV* in contrast to previous PCR analyses. We analyzed the *pheU* and *pheV* loci in strain LM14603/08 by PCR, and did not obtain an amplicon for either site (see Additional file 1), indicating an integration of DNA close to *pheU* and *pheV*. An overview of the sizes of integrated DNA within the three sites is given in Table 2 and is shown schematically in Fig. 1. For the strains LM27558_stx2_ and TS25/08, no contig with *pheV* was detected at the downstream site. Additionally, no contig spanning the complete *pheU* or *pheV* locus including additional DNA was obtained for any strain whereas one contig (contig 22) spanning the entire *selC* locus with additional DNA was obtained for only one strain (17584/1).

**Table 2:** Size of all additional DNA integrated in the three sites *selC*, *pheU* and *pheV*

Strain	*selC*	*pheU*	*pheV*
TS18/08	None	None	> 57,010 bp
LM27558_stx2_	> 5002 bp	> 8453 bp	> 3395 bp
RF1a	> 13,763 bp	> 34,112 bp	None
TS25/08	None	None	> 19,489 bp
LM27564	None	> 3797 bp	> 3686 bp
LM14603/08	> 25,398	> 6437 bp	> 6424 bp
K30	None	> 26,836 bp	None
TS21/08	> 32,214 bp	None	> 13,402 bp
17584/1	50,646 bp	None	> 54,896 bp

In regard to the achieved contig length, we received from the whole genome sequencing procedure we have chosen four strains, 17584/1, RF1a, K30, and TS18/08, and analysed the *selC*-, *pheU*- and/or *pheV*-located nucleotide sequences in more detail and identified the corresponding open reading frames. For strain 17584/1 we received one contig (contig 22) that includes DNA spanning the whole *selC* integration site and two contigs (contig 20 and 35) that include parts of the DNA integrated within *pheV*. For the strains RF1a and K30, we identified two contig each (contig 2 and 30, and contig 16 and 22, respectively) that include parts of the DNA inserted within *pheU*. In addition, two contigs (contig 18 and 39) were also found for the strain TS18/08 including DNA inserted within *pheV*.

### Borders of the nucleotide sequences integrated in tRNA genes

**Fig. 2:**
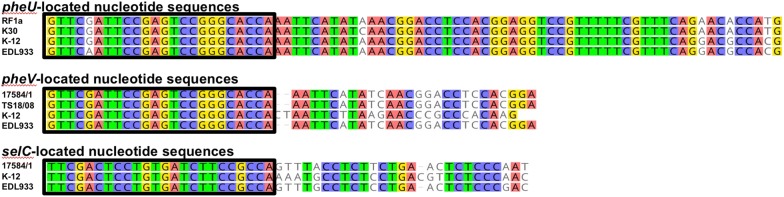
ClustalW alignment of the 5′ termini of the nucleotide sequences adjacent to *pheU*, *pheV* and *selC* tRNA genes. The characters in the boxes are parts of the annotated tRNA genes. Colored characters indicate identities between the nucleotide sequences of the strains

The analysed nucleotide sequences of the strains 17584/1, RF1a, K30 and TS18/08 are integrated downstream of the tRNA genes *pheU*, *pheV* or *selC* and were compared to the sequences of the integration sites of *E. coli* K-12 substr. MG1655 (Acc. No. NC_000913) and EDL933 (Acc. No. NZ_CP008957) (Fig. 2). The tRNA genes *pheU*, *pheV* and *selC* of the analysed strains are similar to the genes of *E. coli* K-12. Differences occur downstream of the tRNA genes especially for the *selC* and with a greater extent for the *pheV* associated insertions.

The insertions stop on the 3′ terminus with a direct repeat (24 bp for *selC*; 22 bp for *pheU* and *pheV*) that is part of the corresponding annotated tRNA gene of *E. coli* K-12. For the direct repeats of *pheU* and *pheV*, a reading frame shift in the analysed sequences was recorded when compared to the *E. coli* K-12 sequences (data not shown).

### Structural characterization of inserted DNA in the integration sites *pheU*, *pheV* or *selC*

*Escherichia coli* O113:H21 strain TS18/08 expresses the toxins SubAB, Cdt-V and Stx [25, 29]. The screening of the LEE-typical insertion sites merely indicates an occupation of the *pheV*-associated insertion site only. Even if we did not receive a contig spanning the whole insertion sequence, we identified two contigs that include the 3′ terminus of *pheV* (38.9 kbp) encoding 45 CDS and the 5′ terminus of the *pheV* corresponding with a direct repeat (16.9 kbp) with 33 CDS (CDS 46–78) (Additional file 1: Figure S2). The CDS have a length between 114 and 9201 bp. With RASTk analysis, 48 CDS were designated as hypothetical proteins. By blastx analyses 34 of the 48 CDS could be confirmed as hypothetical proteins but 14 were characterized as common proteins mostly containing mobile elements (Additional file 1: Table S1).

The second STEC strain that was analysed in detail is RF1a of the serotype O8:H6, which has insertions near *selC* and *pheU*. Since the contig lengths spanning parts covering the *selC* associated nucleotide sequence were low, we only analysed the *pheU*-affiliated parts of contigs 2 (7.3 kbp) and 30 (26.2 kbp) (Additional file 1: Figure S3). The *pheU* 3′ terminus of contig 2 contains four CDS and the downstream *pheU* direct repeat 5′ terminus of contig 30 contains 40 CDS (CDS 5–44) ranging from 120 to 3516 bp (Additional file 1: Table S2). After blastx analyses, 10 CDS are of unknown function, while the remaining CDS could be identified in terms of their encoded protein.

STEC O55:Hnt strain K30 contains foreign DNA only within the *pheU* gene with 20 CDS within the *pheU*-associated part of contig 16 (23.1 kbp) and six CDSs within contig 22 (3.1 kbp) (Additional file 1: Figure S4). The length of the CDS varies between 114 and 5511 bp, and seven of the CDS were of unknown function (Additional file 1: Table S3).

**Fig. 3:**
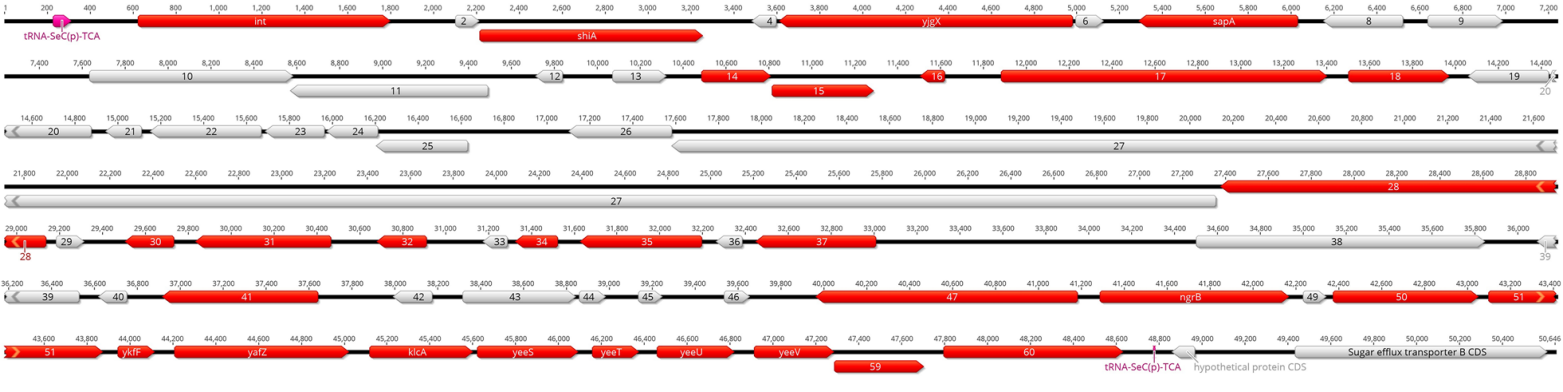
Schematic overview of the *selC*-located part of contig 22 of strain 17584/1. Pink arrows indicate the complete or truncated *selC* tRNA gene. CDS with virulence potential are shown as red arrows with reading direction and correlation to CDS length. The names of some genes are shown. Grey arrows indicate CDS coding for mobile elements, metabolic, fitness factors or hypothetical proteins. The numbers above the arrows indicate base pairs

For O91:H21 strain 17584/1 we could analyse both occupied integration sites *pheV* and *selC*. For *pheV* we found two contigs containing parts of the inserted DNA, contig 20 (32.1 kbp) and contig 35 (21.5 kbp) (Additional file 1: Figure S5), and for *selC* we identified contig 22, including the entire nucleotide sequence located in *selC* (48.5 kbp) of strain 17584/1 (Fig. 3). In contigs 20, 35 and 22 we detected 49, 35 and 60 CDS, respectively. The CDS lengths of the *pheV*-associated DNA ranges between 114 and 3120 bp (Additional file 1: Table S4) and for the *selC* associated DNA between 114 and 9780 bp (Additional file 1: Table S5) with 22 CDS of coding for hypothetical proteins.

### Predicted proteins encoded by DNA insertions in *pheU*, *pheV* or *selC*

The predicted proteins belong to the *ato* system, the type IV toxin-antitoxin system or to the B12 uptake system. Moreover, hemolysins/hemagglutinins, transporters, proteins containing domains of unknown function, methyltransferases, regulators, other hypothetical proteins or proteins belonging to mobile genetic elements were detected (Table 3).

**Table 3:** Predicted proteins encoded by inserted DNA adjacent to *pheV*, *pheU* or *selC*

Proteins	TS18/08	RF1a	K30	17584/1	
	*pheV*	*pheU*	*pheU*	*pheV*	*selC*
Mobile elements	x	x	x	x	x
Transporters					
SapA^a^			x	x	x
ShiA-homolog^a^				x	x
Per-activated serine protease autotransporter enterotoxin EspC				x	
Outer membrane porin OmpF	x			x	
Antigen 43^a^	x			x	
Ato system					
AtoS/AtosC				x	
AtoC				x	
AtoD				x	
AtoA				x	
AtoE				x	
AtoB^a^				x	
Type IV toxin-antitoxin system					
YeeP/NgrB^a^	x	x		x	x
YeeR^a^				x	
YeeS^a^	x	x		x	x
YeeT^a^	x	x		x	x
YeeU^a^	x	x		x	x
YeeV^a^	x	x		x	x
Domains of unknown function					
DUF3987 containing protein		x			
DUF957 containing protein^a^		x	x	x	
DUF4338 containing protein			x		
DUF4222 containing protein	x				
DUF2251 containing protein				x	
DUF1705 containing protein					x
DUF2569 containing protein					x
Methyltransferases					
Z1226^a^	x	x	x	x	x
DNA-cytosine methyltransferase					x
B12 uptake system					
TonB-dependent receptor^a^	x			x	
Outer membrane vitamin B12 receptor BtuB	x			x	
Hemolysin/hemagglutinin					
Putative member of ShlA/HecA/FhaA exoprotein family					x
Putative large exoprotein involved in heme utilization or adhesion of ShlA/HecA/FhaA family	x		x		x
Putative adhesin/hemagglutinin/hemolysin	x				x
RTX toxin activating lysine-acyltransferase	x				
Hemolysin expression modulating protein	x			x	
Hemolysin activator protein precursor^a^					x
Regulators					
DNA-binding proteins^a^	x	x			x
dNTP triphosphohydrolase		x			
Transcriptional regulators^a^		x	x	x	x
HecB-like protein	x				
ProQ/FINO family protein	x				
Others					
Helicases^a^	x	x	x		
YkfF^a^		x		x	x
YafZ^a^	x	x		x	x
Inovirus Gp2 family protein^a^	x	x		x	
Outer membrane protein X precursor	x				
KlcA^a^	x	x		x	x
Z5092^a^		x	x		
EAL domain-containing protein		x			
*N*-acetylgalactosamine 6-sulfate sulfatase (GALNS)	x			x	
Lipoprotein bor	x				
YfjI	x			x	
Ash family protein				x	
Restriction endonuclease					x
Putative phosphoethanolamine transferase^a^	x	x	x	x	x
l-lactate permease	x				
Aec62^a^				x	
Putative competence protein			x		
Hypothetical proteins	x	x	x	x	x

x = putative gene is present in the corresponding tRNA locus

^a^Grouped or partly grouped as virulence factors by RASTk analysis

### Definition of the new genomic island GI*selC*_17584/1_

As mentioned above, we were able to identify the complete foreign DNA insertion adjacent to *selC* of strain 17584/1 by whole-genome sequencing. Calculation of the GC-content of the *selC*-associated nucleotide sequence resulted in 48.4% and thus differs by 2.5% in comparison to the complete genome showing a GC-content of 50.9%. Because we detected the presence of integrases and mobile elements in addition to genes for increased fitness and putative roles in pathogenicity, we could define a new genomic island that was named GI*selC*_17584/1_ (Fig. 3).

**Fig. 4:**
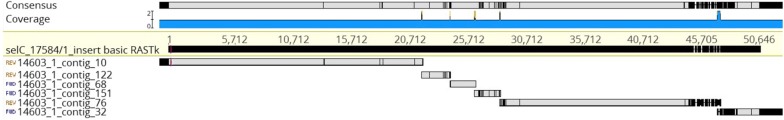
Alignment of *selC* genomic region of strain 17584/1 (yellow highlighted) with contigs of strain 14603/8

A mapping of the contigs of the other sequenced strains using the GI*selC*_17584/1_ as reference (Geneious mapper) identified sequences spanning the GEI in *selC* of strain LM14603/08 within contigs 10, 122, 68, 151, 76 and 32 (see Fig. 4) and parts of the GEI in strain TS21/08 (contigs 3, 52) from pos. 1 to 27,118 (not shown).

### Comparison of GI*selC*_17584/1_ to already described GEIs

**Fig. 5:**
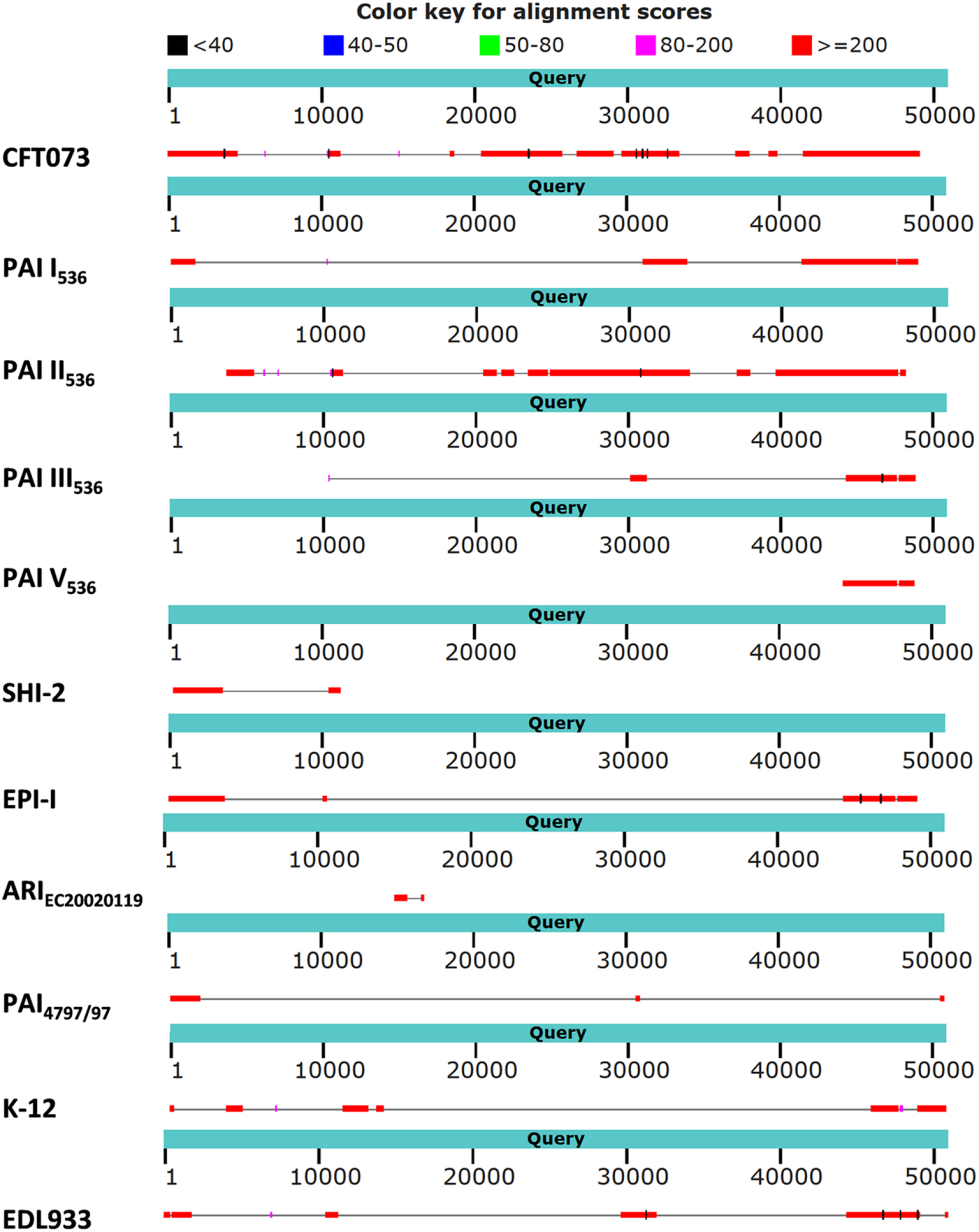
Schematic overview of Blast results for *selC* GEI in comparison with different strains and pathogenicity islands. The query sequence (blue bars) of the new GEI shows information of nucleotide positions and, below that, the allocated sequence of the respective strains or PAIs (grey line) with significant sequence matches (red bars)

Sequence comparison of GI*selC*_17584/1_ with other Enterobacteria revealed in parts similarity to other already described GEIs (see Fig. 5). Parts of GI*selC*_17584/1_ can be found in *E. coli* CFT073 (NC_004431.1), K-12 and EDL933 or in GEI PAI I_536_, PAI II_536_, PAI III_536_ and PAI V_536_ of UPEC 536 (Acc. No. NC_008253), SHI-2 of *Shigella flexneri* strain M90T (Acc No. AF141323), EPI-I of ExPEC strain BEN2908 (Acc. No. AY857617), ARI_EC20020119_ of *E. coli* O157:H7 strain EC20020119 (Acc No. HQ018801) and PAI_4797/97_ of STEC strain 4797/97 (Acc no. AJ278144).

The neighbourhood of *selC* was also detected as integration site for SHI-2 PAI of *Shigella flexneri* strain M90T. The GEI described here shares the first 2877 bp (pos. 338–3215, 99% identity) with SHI-2 including *shiA* (CDS 3). In addition, position 1–3658 bp shares 96.8% sequence identity to a 49.6 kbp-large genomic island EPI-I with an avian background and is described as comprising genes for carbohydrate metabolism, mobility and virulence [30]. Additionally, the direct repeats described in that study were also detected in the GI*selC*_17584/1_ (2× TTCGACTCCTGTGATC at position 299 bp and 48,813 bp (TTTGGGGGTACTTTAGGGGGT at pos. 433 bp and TTTGGGGGTTCTTATGGGGGT at pos. 48,737 bp). Further, eight pairs of direct repeats between 15 and 24 bp were found distributed throughout the GEI (Repeat finder ver. 1, Biomatters Ltd., Geneious software ver. 10.0.7, data not shown).

CDS 5–6 (pos. 3612–5114 bp) from GI*selC*_17584/1_ show homology to a putative membrane-associated, metal-dependent hydrolase (1502 of 1503 bp, 99% identity) or a phosphoethanolamine transferase (1462 of 1489 bp, 98% identity). The locus is also described in PAI II of UPEC strain 536 with the function of an adhesin. The region of GI*selC*_17584/1_ including CDS 10–12 (pos. 7632–9904 bp) is highly similar (100% identity) to the restriction-modification system originally described for *E. coli* strain HK31 (HK31IM Acc. No. X82231). CDS 22 has similarities to a transglutaminase-like enzyme and shows 98% homology with parts of a described antimicrobial resistance island in *E. coli* O157:H7 strain EC20020119 (Acc. No. HQ018801). This island was also identified as a genomic location for the sequence from pos. 14,956 to 15,783 with parts of CDS 21 and 23 of GI*selC*_17584/1_ by Blast comparison.

Other CDS of GI*selC*_17584/1_ (24–28, 35–37, 47–53) were also detected encoded on pathogenicity islands either in PAI I or PAI II of UPEC 536 (Acc. No. NC_008253). Comparison of the GEI with PAIs of UPEC strain 536 shows about 27,000 bp sequence overlap in several fragments and parts of PAI I, II and III within the GEI which is depicted in Fig. 5.

CDS 48, annotated as NgrB, shows 99% homology to a GTPase of the YeeP family and is also described as being encoded in PAI II of UPEC strain 536. CDS 60, annotated as Z1226 with 99% identity to a restriction methylase, is also found in the mentioned strain encoded on PAI V and homologs are found in UPEC strain CFT073. Also CDS 49–60 and the following sequence of the 3′ end with a size of 6581 bp share 99% homology with this UPEC strain.

## Discussion

The results of this study have shown that LEE-negative STEC isolated from different foods contain foreign DNA in the three known LEE integration sites that may contribute to their fitness, potentially resulting in higher adaptation capacity in the host and also supporting their pathogenicity.

We have analysed the genome sequences of nine LEE-negative STEC strains for horizontally-acquired DNA adjacent to the typical LEE integration sites *pheU*, *pheV* and *selC* and found foreign DNA in at least one integration site in each strain. All three analysed integration sites of the strains LM27558_stx2_ and LM14603/08 are occupied. Four strains were analysed in more depth, revealing genetic information for putative virulence and fitness factors at these integration sites. Many CDS encoded hypothetical proteins and all integration sites included mobile elements such as transposases and phage integrases (see Additional file 1: Tables S1–S5). Furthermore, all analysed strains carry genes at their integration sites that presumably encode for proteins with special domains of unknown functions.

The putative restriction methylase Z1226 locus was commonly identified near the 3′ terminus of the corresponding insertion in all strains. Moreover, all analysed integration sites contained the putative phosphoethanolamine transferases YjgX or YhbX.

The following transporter genes were identified in three (TS18/08, K30 and 17584/1) of the four strains. The product of the transporter gene *shiA* shows similarities to a quinone reductase/NADPH oxidoreductase protein [31]. OmpF forms pores in the outer membrane allowing small molecules to diffuse and could also be found in *E. coli* K-12. SapA is a putative ABC transporter. Antigen 43 is an outer membrane protein, autotransporter and a putative adhesion protein that was found in HUS sera [32–34]. The Per-activated serine protease autotransporter enterotoxin EspC, present in *pheV* of strain 17584/1, contains the superfamily Peptidase_S6 motif and is also designated as a hemoglobin-binding protease (hbp) (EGW81517), serine protease (WP_001367507), autotransporter (KLH79608), autotransporter outer membrane beta-barrel domain containing protein (ANE59238), serine protease pic autotransporter (EHI35518) or serine protease SepA autotransporter precursor (BAX13941). Peptidase_S6 is present in many serine proteases as Hbp, EspP, Pet, EatA, EspC or Pic from *E. coli* or EspA from *Shigella flexneri.* In these proteins the Peptidase_S6 domain is combined with an autotransporter domain. The autotransporter domain is missing in the Per-activated serine protease autotransporter enterotoxin EspC analysed here. The gene is located at the 3′ terminus of contig 20 and the autotransporter domain was not captured on the same contig. To prove this we performed a PCR and amplified the putative whole gene, including the autotransporter domain and the signal peptide sequence. With the receipt of an amplificate length of 3993 bp, we could confirm the presence of the entire Per-activated serine protease autotransporter enterotoxin EspC gene in strain 17584/1 (see Additional file 1: Figure S6).

The *pheV*-adjacent foreign DNA of strain 17584/1 carries an *ato* system that was recently described in the Locus of Adhesion and Autoaggregation (LAA) pathogenicity island [35]. AtoS (sensor kinase) and AtoC (response regulator) belong to a two-component regulatory system that stimulates the expression of the *atoDAEB*-operon in the presence of acetoacetate or spermidine [36–38]. The *atoDAEB*-operon is fundamental for cellular processes such as short-chain fatty acid catabolism, poly-(R)-3-hydroxybutyrate biosynthesis and chemotaxis [39].

Surprisingly, three (TS18/08, RF1a, 17584/1) of the four strains encode a toxin-antitoxin gene pair (*yeeV*/*yeeU*). The toxin usually binds to an essential enzyme in the cell and inhibits the enzymatic activity. The antitoxin binds the toxin and restores viability. Growth inhibition was detected and could be restored by antitoxins but the physiological role is still under investigation. For chromosomally encoded toxin-antitoxin systems, two models for cellular function and role have been proposed: The first leads to programmed cell death in response to starvation by transcriptional attenuation using the toxin-antitoxin system and therefore providing nutrients for the remaining population [40]. The second function is to modulate the rate of metabolic processes in response to environmental stress [41]. The genes for YeeP/NgrB (50S ribosome-binding GTPase), YeeR (inner membrane protein), YeeS (metallopeptidase) and YeeT (unknown function) are located in the same direction upstream of *yeeU* and *yeeV*. The toxin-antitoxin system was first described within the cryptic prophage CP4–44.

A vitamin B12 uptake system was detected in both *pheV*-integrated DNA segments in the strains TS18/08 and 17584/1.

Two strains carry genes encoding for hemagglutinin or hemolysin, both which are part of GI*selC*_17584/1_. Blast comparison of CDS 28, the annotated hemolysin activator protein precursor (two partner secretion) family, shows sequence homologies (1732 of 1767 bp) to *cdiB* of the contact-dependent growth inhibition (CDI) system. This system is used by bacteria to express two-partner secretion proteins encoded by *cdiA* and *cdiB* to bind to BamA in the outer membranes of target cells and inhibit their growth [42, 43]. The CDI system was found in different genomic and pathogenicity islands and analysed in detail in uropathogenic *E. coli* [44]. CDS 25 with homology to an adhesin within the CDI system and CDS 27 annotated as CdiA, secreted exoprotein with conserved domains of hemagglutination activity, are both components of the CDI system. Annotated CDS 20 and CDS 23 are also both similar to hemagglutinin when compared to the NCBI database with Blastx. Furthermore, in *pheV* of TS18/08 the *hha* homologous gene for a hemolysin-expressing modulating protein is present, which downregulates expression of hemolysin in a complex with H-NS in *E. coli* O6:H1. The RTX toxin activating lysin-acetyltransferase, also termed Hemolysin C (HlyC), converts HlyA to an active toxin.

All analysed strains contain regulators that may control the expression of the surrounding genes or of more distantly encoded genes. The ProQ/FinO family is involved in the control of F plasmid transfer [45].

There are additional proteins that are not characteristically grouped but are mostly present in the analysed strains as, for example, the anti-restriction protein KlcA described by Serfiotis-Mitsa and coworkers [46], which is often encoded on plasmids, conjugative transposons and phages and is supposed to increase the chances of entering a new bacterial host due to a Type I DNA restriction and modification (RM) system, which would usually destroy the invading DNA [46]. YfjI, YkfF (DUF905 domain containing protein) and YafZ are described in *E. coli* K-12 with unknown protein functions. As YkfF and YafZ, the inovirus Gp2 family proteins were partially grouped to the virulence factors by RASTk analysis but their function is still unknown. Z5092 encodes an uncharacterised RNA-directed DNA polymerase. The EAL domain is found in signaling proteins [47]. The lambda phage lipoprotein Bor is an outer membrane protein of *E. coli* and confers serum resistance [48].

The GI*selC*_17584/1_ was partially found in another characterised, already published, strain. Bertin and coworkers described an altered *selC* locus with CP4-int at the 3′ terminus and similarities to the locus of proteolysis activity (Acc No. AJ278144 strain 4797/97 STEC) and SHI-2 (Acc No. AF141323 strain M90T *S. flexneri*) and demonstrated that the *selC* locus is frequently used as an integration site for PAIs with CP4 integrase genes [49]. Homologies to CP-4 phage-derived proteins were also found in the GI*selC*_17584/1_. The described genomic island is of mosaic composition and harbours several already described parts from other genomic/pathogenicity islands. Several parts of UPEC strain 536 were found throughout the GEI and the last approx. 6000 bps at the 3′ terminus show homologies to UPEC strain CFT073 (see Fig. 5). The complete sequence of the GEI is only found in STEC strains 117 and 453 (Acc. No. NZ_MPGS01000001.1 and NZ_MPGR01000001.1) isolated from deer feces in Finland with 99% similarity each, which may hint at a recent clonal distribution of the GEI. Large parts were also detected in two *E. coli* strains FHI98 and FHI29 from human feces in Norway (Acc. No. LM997367.1 and Acc. No. LM995856.1). The mosaic structure of the GI*selC*_17584/1_ with sequences partly described in several different other genomic islands illustrates again the high genome plasticity within the *E. coli* species independent of the different pathotypes. We can only speculate on the role of this additional DNA adjacent to *selC* in foodborne strain 17584/1 and others. Since many of the already described protein functions deal with increased fitness, response to environmental stress or adhesion and fine-tuning of the metabolism, we suppose this genomic island is a collection of DNA and functions as a survival kit for the bacterium in unfavorable environmental conditions.

In conclusion, we detected foreign DNA in the well-known LEE integration sites *pheU*, *pheV* and *selC* in different LEE-negative STEC strains. Some annotated CDS located in these integration sites can be designated as virulence factors. However, whether foreign DNA in the different integration sites supports virulence or pathogenicity to the respective strains or whether the occupation of the integration sites hinders the reception of other high impact pathogenicity islands like the LEE and is, therefore, a cause for decreased pathogenicity will be an interesting question for future studies.

## Additional file

**Additional file 1.** Additional Figures S1–S6 and Tables S1–S4.

## Abbreviations

LEE: Locus of Enterocyte Effacement
STEC: Shiga toxin-producing *E. coli*
GEI: genomic island
PAI: pathogenicity island
EHEC: enterohemorrhagic *E. coli*
UPEC: uropathogenic *E. coli*
nt: not typeable
nm: non motile
DUF: domain of unknown function
CDS: coding DNA sequence
