# Supplementary material for: Determination of virulence and fitness genes associated with the pheU, pheV and selC integration sites of LEE-negative food-borne Shiga toxin-producing Escherichia coli strains
Source: Gut Pathog. 2018 Oct 8;10:43. doi: 10.1186/s13099-018-0271-8 (PMC6174562; doi:10.1186/s13099-018-0271-8)
Supplement: Supplementary file 1 — Additional file 1. Additional Figures S1–S6 and Tables S1–S4. [file 13099_2018_271_MOESM1_ESM.pdf]

Additional File 1

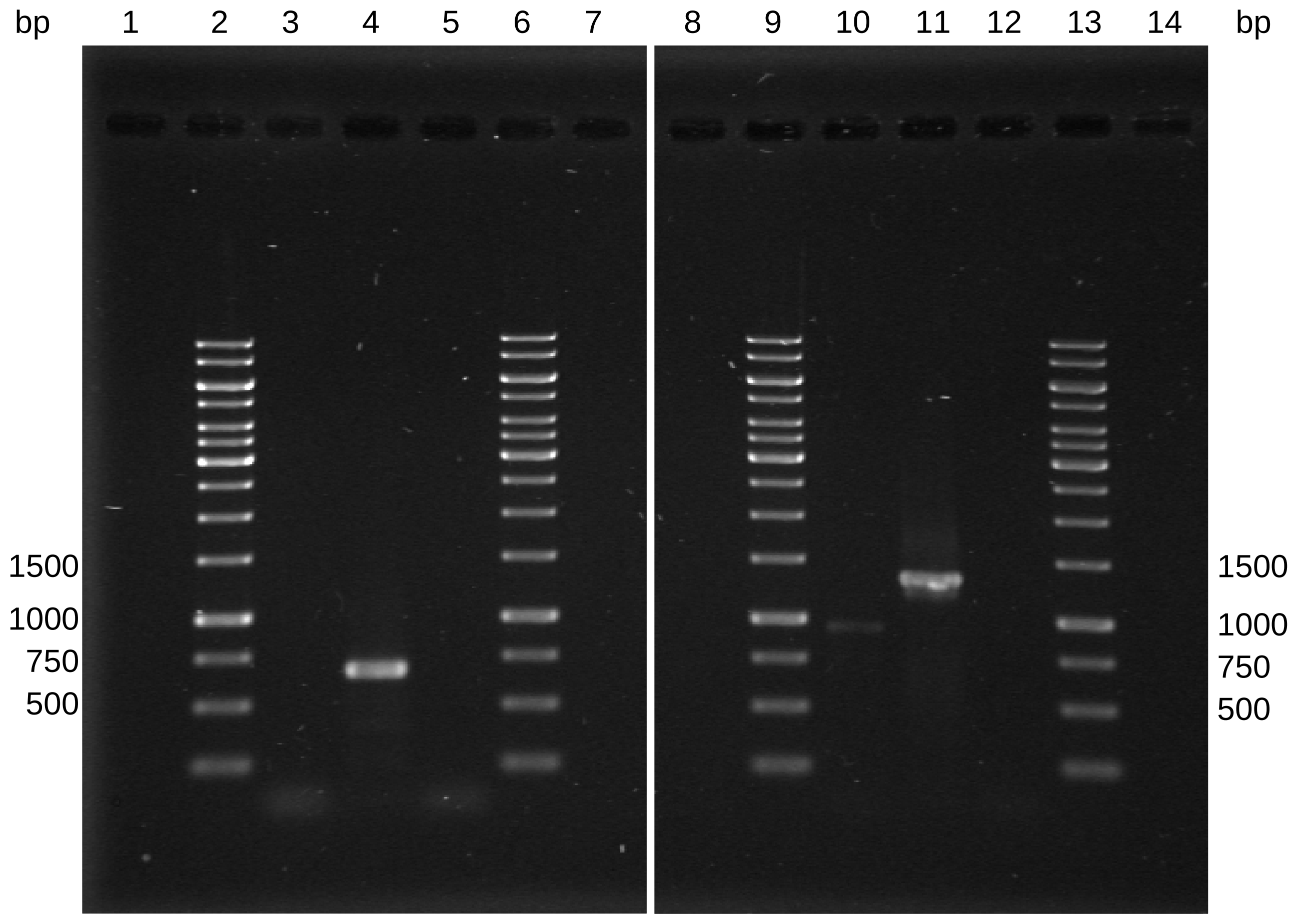

Figure S1: PCR detection of DNA insertions in integration sites *pheU* (left agarose gel) and *pheV* (right agarose gel) of strain LM 14603/08 (lane 3 and 10, respectively). Amplification sizes of 664 basepairs (bp) and 1306 bp indicated intact (no DNA insertion) *pheU* and *pheV* integration sites, respectively, as shown for strain TS18/08 (lane 4) and RF1a (lane 11). Lane 5 and 12 are the negative controls, lane 2, 6, 9 and 13 include GeneRuler 1 kb DNA ladder from Thermo Scientific. Lane 1, 7, 8 and 14 are empty.

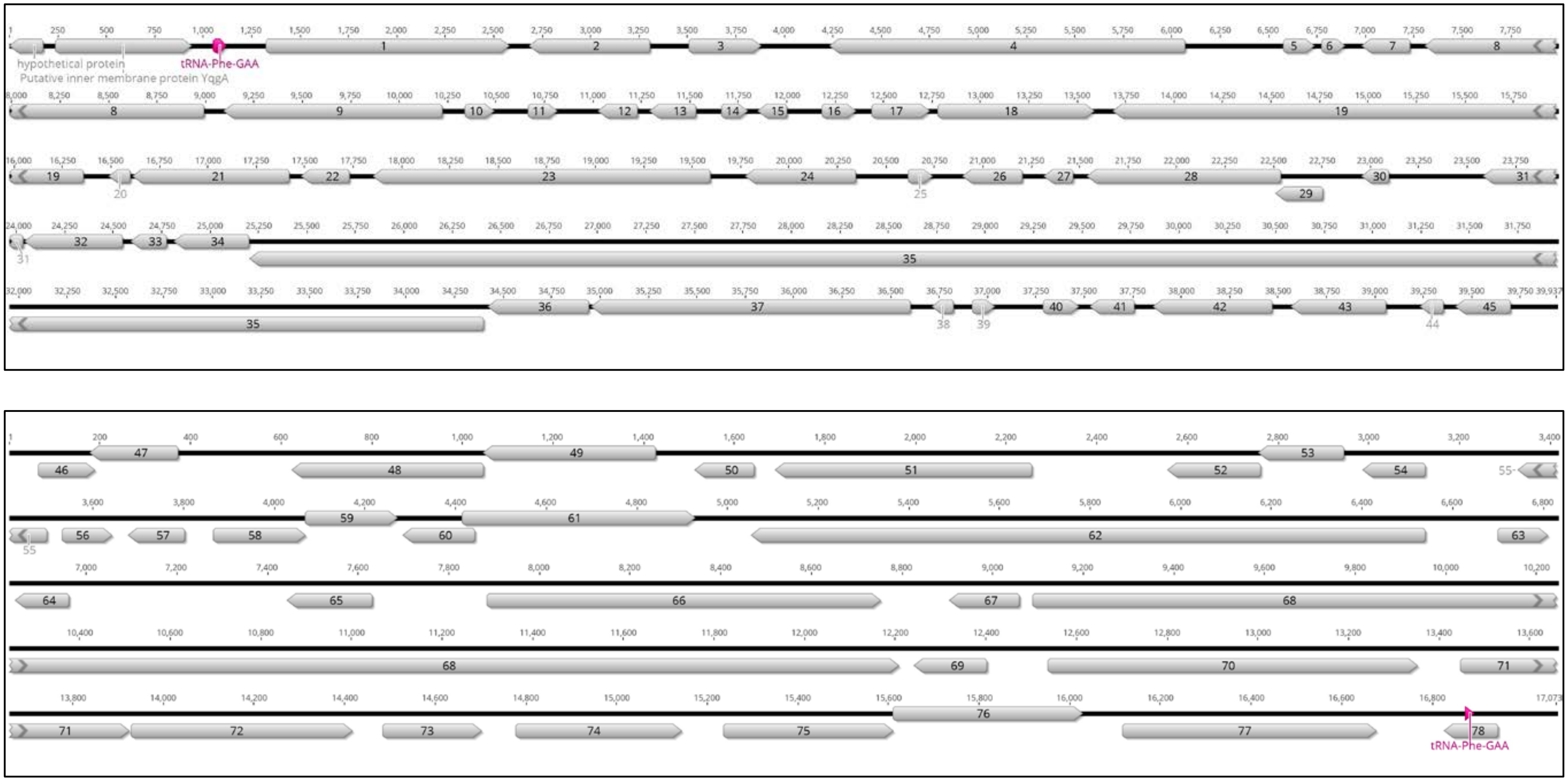

Figure S2. Schematic overview of the *pheV*-located parts of contig 39 (above) and 18 (below) of strain TS18/08. Pink arrows indicate the complete or truncated *pheV* tRNA gene. Grey arrows indicate annotated CDS with reading direction and correlation to CDS length. Numbers above the arrows indicate base pairs.

Table S1. Annotation and coding sequences (CDS), gene sequence length and direction of the *pheV* tRNA gene associated part of contig 39 (CDS 1-45) and 18 (CDS 46-78) of strain TS18/08.

CDS	Function		Start	Stop	Length [bp]	Strand[a]	Group[b]
	RASTk annotation	Blastx analysis of hypothetical proteins (coverage/identity (%); E-value; Accession No.)					
	hypothetical protein		181	2	180	-	2
	Putative inner membrane protein YqgA		240	947	708	+	3
	tRNA-Phe-GAA		1053	1128	75	+	tRNA
1	Integrase		1326	2588	1263	+	4
2	Putative phosphoethanolamine transferase YhbX		3317	2685	633	-	3
3	TonB-dependent receptor		3508	3888	381	+	4
4	Outer membrane vitamin B12 receptor BtuB		6078	4234	1845	-	3
5	hypothetical protein	Transposase (100/100; 9e-34; WP_077904407.1)	6576	6743	168	+	1
6	hypothetical protein	[c]	6775	6903	129	+	2
7	hypothetical protein	[c]	7236	6979	258	-	2
8	N-acetylgalactosamine 6-sulfate sulfatase (GALNS)		9002	7305	1698	-	3
9	Outer membrane porin OmpF		10233	9097	1137	-	3
10	hypothetical protein	[c]	10340	10498	159	+	2
11	hypothetical protein	lipoprotein bor (30/94; WP_095764448.1; 0.98)	10665	10823	159	+	3
12	hypothetical protein	Transposition helper protein (70/86; 6e-11; OSK24662.1)<br>Mobile element protein (70/86; 6e-11; KGI47791.1)<br>IS66 family element (70/86; 8e-11; EIH91436.1)<br>Putative transposase (93/53; 2e-09; KDY73070.1)<br>Effector protein (91/59; 4e-09; ETE13342.1)<br>ISPsy5 (91/59; 4e-09; EFW61180.1)	11238	11026	213	-	1
13	hypothetical protein	Phage protein (70/45; 6e-10; WP_023277533.1)	11541	11296	246	-	2
14	hypothetical protein	[c]	11664	11807	144	+	2
15	hypothetical protein	[c]	12008	11850	159	-	2
16	hypothetical protein	[c]	12183	12365	183	+	2
17	hypothetical protein (probably bogus)	Transposase (99/100; 3e-64; WP_000088311.1)	12441	12743	303	+	4
18	Mobile element protein		12779	13597	819	+	4
19	DNA helicase		16364	13683	2682	-	4

20	hypothetical protein	c	16608	16492	117	-	2
21	HecB-like protein		17430	16615	816	-	3
22	FIG00643706: hypothetical protein	c	17739	17485	255	-	2
23	FIG00643591: hypothetical protein	DNA helicase UvrD (99/100; 0.0; WP_072127289.1)	19600	17855	1746	-	4
24	Outer membrane protein X precursor		20353	19778	576	-	3
25	hypothetical protein	c	20614	20748	135	+	2
26	putative adhesin/hemagglutinin/hemolysin		21211	20897	315	-	3
27	hypothetical protein	c	21476	21315	162	-	2
28	Putative large exoprotein involved in heme utilization or adhesion of ShlA/HecA/FhaA family		22545	21538	1008	-	3
29	Hemolysin		22762	22511	252	-	3
30	hypothetical protein	Cysteine-rich CPCC (93/80; 2e-19; SET02225.1) Membrane protein (95/69; 4e-14; WP_002087153.1)	23105	22959	147	-	3
31	hypothetical protein	c	24041	23586	456	-	2
32	Putative large exoprotein involved in heme utilization or adhesion of ShlA/HecA/FhaA family		24555	24046	510	-	3
33	hypothetical protein	c	24784	24590	195	-	2
34	hypothetical protein	c	25209	24814	396	-	2
35	Putative large exoprotein involved in heme utilization or adhesion of ShlA/HecA/FhaA family		34406	25206	9201	-	3
36	RTX toxin activating lysine-acyltransferase (EC 2.3.1.-)		34949	34422	528	-	3
37	HecB-like protein		36611	34959	1653	-	3
38	FIG00644081: hypothetical protein	Transposase (80/88; 1e-12; EZC68205.1)	36834	36715	120	-	1
39	hypothetical protein		36925	37044	120	+	2
40	FIG00641264: hypothetical protein	DUF4222 domain-containing protein (98/100; 2e-29; WP_001287885.1)	37287	37478	192	+	2
41	FIG00641476: hypothetical protein	c	37764	37531	234	-	4
42	FIG00640120: hypothetical protein	DNA-binding protein (99/99; 7e-152; WP_096695682.1)	38480	37857	624	-	4
43	FIG00641784: hypothetical protein	Inovirus Gp2 family protein (99/78; 3e-89; WP_001167436.1)	39066	38569	498	-	4
44	FIG00639802: hypothetical protein	c	39363	39235	129	-	2
45	FIG00641015: hypothetical protein	Inovirus Gp2 family protein (98/100; 5e-64; WP_078163266.1)	39706	39416	291	-	3

		DUF3296 domain-containing protein (98/100; 5e-64; PAU30381.1)					
46	Mobile element protein		65	193	129	+	1
47	FIG00640323: hypothetical protein	Inovirus Gp2 family protein (98/98; 7e-41; WP_000553835.1) DUF3296 domain-containing protein (95/86; 4e-32; WP_086524882.1) Transposase (77/88; 7e-25; OJZ36238.1) CP4-6 prophage protein (77/82; 2e-22; AQZ78107.1)	375	178	198	-	1
48	orf, conserved hypothetical protein	c	1049	624	426	-	4
49	FIG00639161: hypothetical protein	c	1429	1046	384	-	4
50	hypothetical protein	c	1647	1513	135	-	2
51	Intergenic-region protein		2259	1690	570	-	4
52	hypothetical protein	c	2765	2556	210	-	2
53	hypothetical protein	c	2949	2758	192	-	2
54	Haemolysin expression modulating protein		3127	2987	141	-	4
55	hypothetical protein	L-lactate permease (34/90; 0.036; WP_072146917.1)	3502	3329	174	-	3
56	FIG00643086: hypothetical protein	c	3532	3645	114	+	2
57	hypothetical protein	c	3806	3678	129	-	2
58	FIG00638000: hypothetical protein	c	3865	4071	207	+	4
59	FIG00638000: hypothetical protein	c	4068	4274	207	+	4
60	FIG00640183: hypothetical protein	c	4446	4285	162	-	2
61	FIG00638808: hypothetical protein	c	4414	4932	519	+	4
62	Uncharacterized protein YfjI		6543	5053	1491	-	3
63	hypothetical protein	c	6701	6814	114	+	2
64	FIG00641572: hypothetical protein	c	6966	6844	123	-	2
65	hypothetical protein	c	7634	7443	192	-	2
66	Uncharacterized protein YeeP		7885	8757	873	+	4
67	hypothetical protein	c	9063	8905	159	-	2
68	Antigen 43		9089	12211	3123	+	4
69	hypothetical protein	c	12405	12241	165	-	2
70	UPF0380 proteins YafZ and homologs		12538	13356	819	+	4
71	Antirestriction protein KlcA		13447	13929	483	+	4
72	UPF0758 protein YeeS		13930	14421	492	+	4
73	Uncharacterized protein YeeT		14484	14705	222	+	4
74	YeeU protein (antitoxin to YeeV)		14779	15147	369	+	4
75	YeeV toxin protein		15236	15613	378	+	4

76	FIG00640476: hypothetical protein	[c]	15610	16032	423	+	4
77	Z1226 protein		16117	16680	564	+	4
78	hypothetical protein	[c]	16948	16826	123	-	2
	tRNA-Phe-GAA		16874	16895	22	+	tRNA

[a] CDS reading direction: + forward, - reverse

[b] assigned groups for predicted CDS function: 1) mobility, 2) hypothetical/ unknown, 3) metabolism/ fitness, 4) virulence factor

[c] no further classification of the hypothetical protein by blastx analysis possible

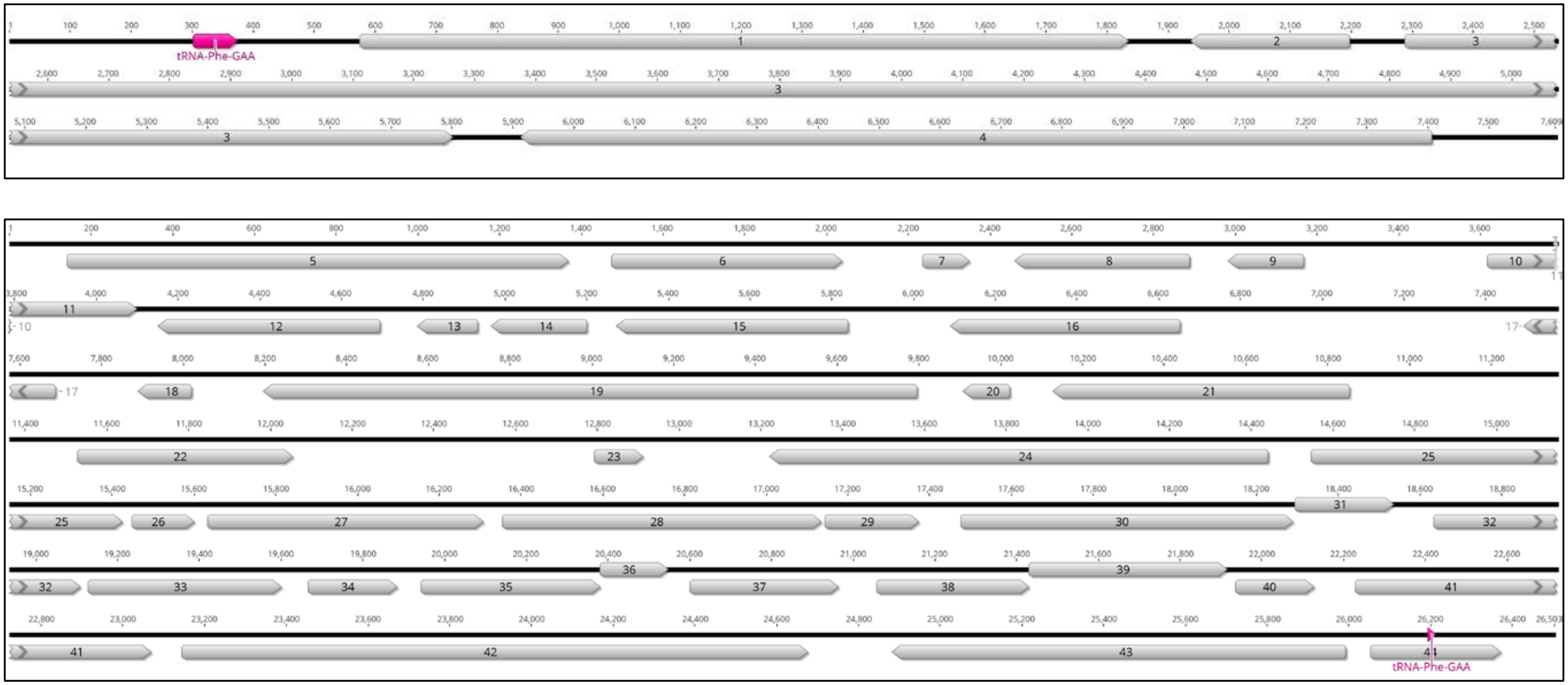

Figure S3. Schematic overview of the *pheU*- located parts of contig 2 (above) and 30 (below) of strain RF1a. Pink arrows indicate the complete or truncated *pheU* tRNA gene. Grey arrows indicate annotated CDS with reading direction and correlation to CDS length. Numbers above the arrows indicate base pairs.

Table S2. Annotation and coding sequences (CDS), gene sequence length and direction of the *pheU* tRNA gene associated part of contig 2 (CDS 1-4) and 30 (CDS 5-44) of strain RF1a.

CDS	Function		Start	Stop	Length [bp]	Strand[a]	Group[b]
	RASTk annotation	Blastx analysis of hypothetical proteins (coverage/identity (%); E-value; Accession No.)					
	tRNA-Phe-GAA		302	377	76	+	tRNA
1	Integrase		575	1837	1262	+	4
2	hypothetical protein	[c]	2200	1937	264	-	2
3	Putative superfamily I DNA helicases		2289	5804	3516	+	4
4	Putative phosphoethanolamine transferase YjgX		7409	5913	1497	-	4
5	dNTP triphosphohydrolase, broad substrate specificity		141	1373	1233	+	3
6	hypothetical protein	Putative membrane protein (97/99; 3e-135; EGI35315.1)	1473	2042	570	+	3
7	hypothetical protein	[c]	2235	2354	120	+	2
8	DNA-binding protein H-NS homolog		2893	2459	435	-	3
9	hypothetical protein	[c]	3171	2983	189	-	2
10	FIG00330653: hypothetical protein	osmoprotectant transport activator ProQ (98/98; 5e-33; WP_087086758.1)	3616	3789	174	+	3
11	FIG00640804: hypothetical protein	prop effector (99/99; 6e-45; WP_089648304.1) proQ/FINO family protein (94/97; 9e-42; OWW54441.1)	3786	4103	318	+	3
12	FIG00641784: hypothetical protein	Inovirus Gp2 family protein (92/99; 2e-119; WP_089691900.1)	4698	4150	549	-	3
13	FIG00641314: hypothetical protein	Putative regulatory protein (78/68; 9e-08; EMX15537.1) Putative membrane protein (62/72; 2e-05; KDU01312.1)	4938	4786	153	-	3
14	hypothetical protein	AlpA family transcriptional regulator (98/100; 1e-51; WP_000958094.1) Prophage CP4-57 regulatory protein family	5203	4967	237	-	4

		protein (98/97; 2e-50; EGW88643.1) Transcriptional regulator (93/96; 6e-47; PAU11715.1)					
15	FIG00639802: hypothetical protein		5844	5272	573	-	4
16	FIG01048891: hypothetical protein	Inovirus Gp2 family protein (99/100; 3e-141; WP_001126818.1)	6657	6091	567	-	4
17	hypothetical protein	c	7691	7494	198	-	2
18	hypothetical protein	c	8023	7889	135	-	2
19	hypothetical protein	DUF3987 domain-containing protein (99/100; 0.0; WP_032082728.1)	9800	8196	1605	-	2
20	hypothetical protein	c	10026	9907	120	-	2
21	Intergenic-region protein		10858	10127	732	-	4
22	hypothetical protein	DNA-binding protein (99/99; 1e-92; WP_096006760.1)	11528	12058	531	+	3
23	hypothetical protein	c	12794	12916	123	+	2
24	FIG00639329: hypothetical protein	c	14445	13222	1224	-	4
25	NgrB		14546	15430	885	+	4
26	FIG00638087: hypothetical protein	c	15449	15604	156	+	2
27	FIG00641766: hypothetical protein	Transcriptional regulator (99/100; 1e-164; WP_032082725.1) WYL domain-containing protein (99/99; 6e-164; WP_096973699.1)	15633	16313	681	+	4
28	FIG00637880: hypothetical protein	Hypothetical membrane protein (99/99; 0.0; EGI47989.1)	16356	17138	783	+	4
29	UPF0401 protein YkfF		17144	17377	234	+	4
30	UPF0380 proteins YafZ and homologs		17476	18294	819	+	4
31	Antirestriction protein klcA		18294	18539	246	+	4
32	Antirestriction protein klcA		18633	19112	480	+	4
33	UPF0758 protein YeeS		19128	19604	477	+	4
34	Uncharacterized protein YeeT		19667	19888	222	+	4
35	hypothetical protein	Antitoxin of toxin-antitoxin stability system (99/99; 6e-107; WP_096123738.1)	19944	20384	441	+	3
36	FIG00642070: hypothetical protein	Antitoxin of the YeeV-YeeU toxin-antitoxin system (98/98; 1e-30; EIO23170.1)	20381	20551	171	+	4
37	YeeU protein (antitoxin to YeeV)		20601	20969	369	+	4

38	YeeV toxin protein		21059	21433	375	+	4
39	Z5092 protein		21430	21918	489	+	4
40	FIG00640476: hypothetical protein	DUF957 domain-containing protein (98/100; 4e-33; WP_032082787.1) Z1225 protein (98/89; 8e-30; CAD33791.1)	21935	22132	198	+	4
41	Z1226 protein		22229	23074	846	+	4
42	FIG00641524: hypothetical protein	EAL domain-containing protein (99/100; 0.0; WP_001609560.1)	23145	24680	1536	+	3
43	Transposase InsL for insertion sequence element IS186		25995	24883	1113	-	1
44	hypothetical protein	ISAfe1%2c transposase (45/94; 2e-22; CSO99818.1)	26053	26376	324	+	1
	tRNA-Phe-GAA		26195	26216	22	+	tRNA

[a] CDS reading direction: + forward, - reverse

[b] assigned groups for predicted CDS function: 1) mobility, 2) hypothetical/ unknown, 3) metabolism/ fitness, 4) virulence factor

[c] no further classification of the hypothetical protein by blastx analysis possible

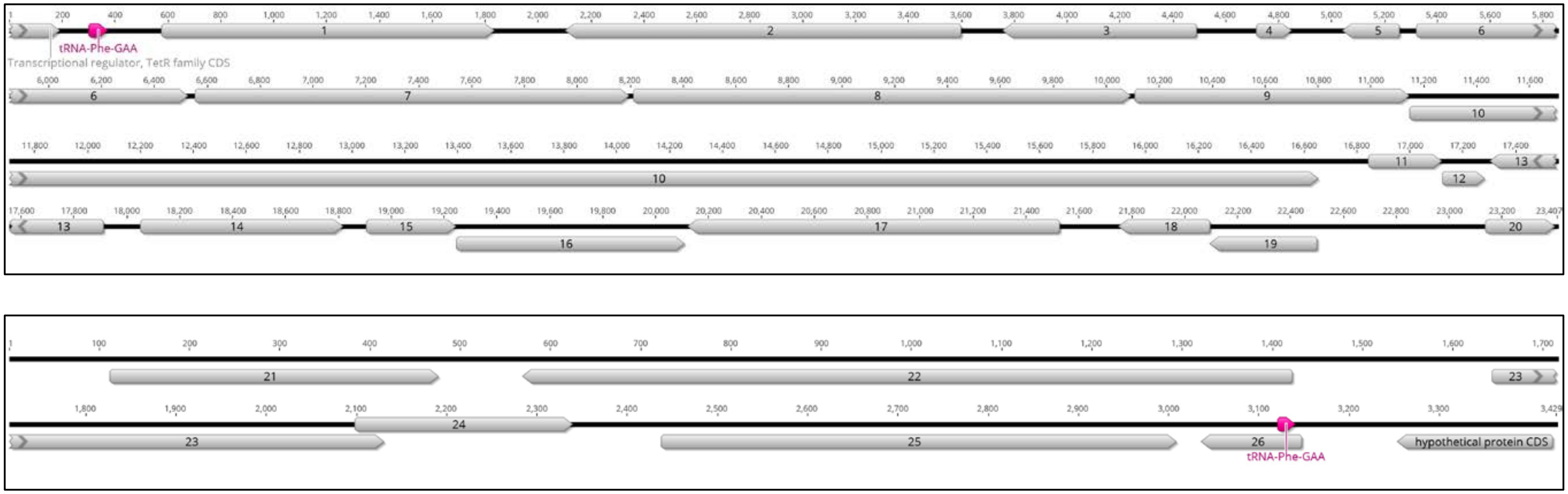

Figure S4. Schematic overview of the *pheU*- located parts of contig 16 (above) and 22 (below) of strain K30. Pink arrows indicate the complete or truncated *pheU* tRNA gene. Grey arrows indicate annotated CDS with reading direction and correlation to CDS length. Numbers above the arrows indicate base pairs.

Table S3. Annotation and coding sequences (CDS), gene sequence length and direction of the *pheU* tRNA gene associated part of contig 16 (CDS 1-20) and 22 (CDS 21-27) of strain K30.

CDS	Function		Start	Stop	Length [bp]	Strand[a]	Group[b]
	RASTk annotation	Blastx analysis of hypothetical proteins (coverage/identity (%); E-value; Accession No.)					
	Transcriptional regulator YjdC, AcrR family		1	195	195	+	3
	tRNA-Phe-GAA		302	377	76	+	tRNA
1	Integrase		575	1837	1263	+	4
2	Putative phosphoethanolamine transferase YjgX		3604	2102	1503	-	4
3	Peptide transport periplasmic protein sapA (TC 3.A.1.5.5)		4496	3756	741	-	4
4	Integrase		4716	4853	138	+	1
5	hypothetical protein	[c]	5263	5042	222	-	2
6	hypothetical protein	DUF4338 domain-containing protein (99/100; 0.0; WP_000549798.1)	5320	6534	1215	+	2
7	putative competence protein		6556	8202	1647	+	3
8	hypothetical protein	[c]	8214	10097	1884	+	2
9	hypothetical protein	[c]	10109	11152	1044	+	2
10	Probable ATP-dependent helicase Lhr		11149	16659	5511	+	3
11	hypothetical protein	[c]	16845	17126	282	+	2
12	hypothetical protein	[c]	17123	17290	168	+	2
13	Transposase InsF for insertion sequence IS3 @ Mobile element protein		17918	17304	615	-	4
14	Mobile element protein		18052	18822	771	+	4
15	Transposase		18907	19251	345	+	1
16	Transposase		19248	20114	867	+	1
17	Mobile element protein		21533	20124	1410	-	1
18	Mobile element protein		22100	21753	348	-	1
19	hypothetical protein	IS66 family sequence hypothetical protein (99/99; 4e-80; WP_000443282.1) Transposase (99/99; 7e-80; OOJ16471.1)	22507	22097	411	-	1
20	putative regulator PapX protein		23137	23406	270	+	3

21	Transposase InsC for insertion element IS2		112	477	366	+	4
22	Putative large exoprotein involved in heme utilization or adhesion of ShlA/HecA/FhaA family		1423	569	855	-	3
23	Z5092 protein		1644	2132	489	+	4
24	FIG00640476: hypothetical protein	DUF957 domain-containing protein (82/99; 6e-42; WP_001110769.1) Z1225 protein (82/97; 1e-40; CAD33791.1) Aec78 (82/93; 4e-37; AQZ79575.1)	2099	2341	243	+	4
25	Z1226 protein		2438	3010	573	+	4
26	hypothetical protein	[c]	3149	3036	114	-	2
27	hypothetical protein	[c]	3427	3254	174	-	2
	tRNA-Phe-GAA		3121	3142	22	+	tRNA
	Hypothetical protein CDS		3427	3254	174	-	2

[a] CDS reading direction: + forward, - reverse

[b] assigned groups for predicted CDS function: 1) mobility, 2) hypothetical/ unknown, 3) metabolism/ fitness, 4) virulence factor

[c] no further classification of the hypothetical protein by blastx analysis possible

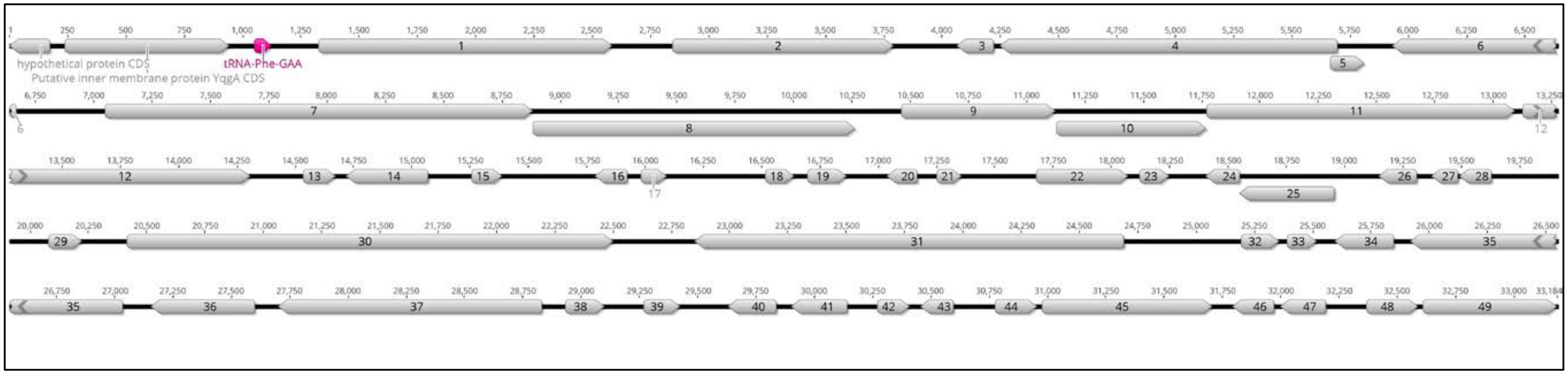

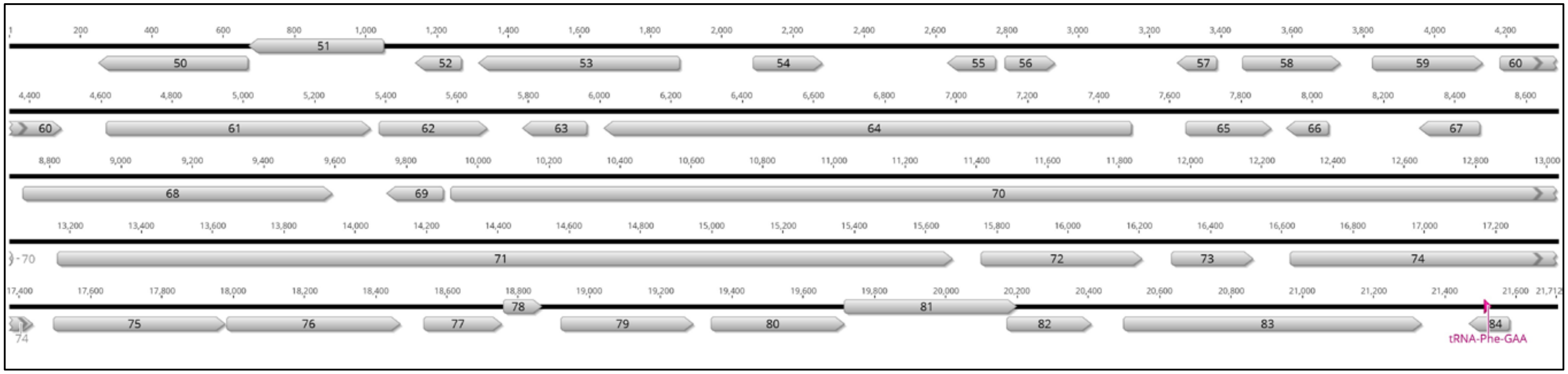

Figure S5. Schematic overview of the *pheV*- located parts of contig 20 (above) and 35 (below) of strain 17584/1. Pink arrows indicate the complete or truncated *pheV* tRNA gene. Grey arrows indicate annotated CDS with reading direction and correlation to CDS length. Numbers above the arrows indicate base pairs.

Table S4. Annotation and coding sequences (CDS), gene sequence length and direction of the *pheV* tRNA gene associated part of contig 20 (CDS 1-49) and 35 (CDS 50-84) of strain 17584/1.

CDS	Function		Start	Stop	Length [bp]	Strand[a]	Group[b]
	RASTk annotation	Blastx analysis of hypothetical proteins (coverage/identity (%); E-value; Accession No.)					
	hypothetical protein		181	2		-	2
	Putative inner membrane protein YqgA		240	947		+	3
	tRNA-Phe-GAA		1053	1128	76	+	tRNA
1	Integrase		1326	2591	1266	+	4
2	ShiA homolog		2847	3797	951	+	4
3	FIG00641916: hypothetical protein	Inner membrane protein YhbX (98/94; 5e-29; EGB65631.1) Phosphoethanolamine transferase (90/63; 2e-10; WP_074169234.1) Hydrolase (90/63; 2e-10; CTU18000.1) Phosphate starvation-inducible protein PsiE (90/63; 8e-10; KHH50094.1) Alkaline phosphatase (90/63; 1e-09; SJG73921.1) Sulfatase family protein (90/63; 3e-09; EMV34095.1)	4226	4065	162	-	3
4	Putative phosphoethanolamine transferase YjgX		5703	4249	1455	-	4
5	FIG00641722: hypothetical protein	Phosphoethanolamine tranferase (56/100, 2e-10; WP_097739339.1) Membrane-associated metal-dependent hydrolase (56/100; 2e-10; ETF17190.1) Arylsulfatase (56/100; 2e-10; ESD40419.1)	5666	5815	150	+	3
6	Peptide transport periplasmic protein SapA (TC 3.A.1.5.5)		6673	5933	741	-	4
7	Signal transduction histidine-protein kinase AtoS (EC 2.7.13.3)		7049	8887	1839	+	3
8	Acetoacetate metabolism regulatory protein AtoC		8884	10269	1386	+	3
9	Acetyl-CoA:acetoacetyl-CoA transferase, alpha subunit (EC 2.8.3.8)		10465	11127	663	+	3

10	Butyrate-acetoacetate CoA-transferase subunit B (EC 2.8.3.9)		11127	11777	651	+	3
11	Short-chain fatty acids transporter		11774	13096	1323	+	3
12	Acetyl-CoA acetyltransferase (EC 2.3.1.9)		13127	14311	1185	+	4
13	hypothetical protein	IS630 family transposase (62/57; 0.46; WP_070082820.1)	14534	14677	144	+	1
14	Mobile element protein		15077	14721	357	-	1
15	FIG00643867: hypothetical protein	Transposase (86/100; 1e-20; KHG91905.1)<br>Cytochrome o ubiquinol oxidase (93/90; 2e-20; EHW83623.1)<br>Protein encoded within IS (86/100; 4e-19; CSQ87570.1)	15258	15392	135	+	1
16	hypothetical protein	IS66 family insertion sequence hypothetical protein (95/85; 0,004; WP_032215269.1)<br>Transposase (56/67; 0.036; WP_072200196.1)<br>Helix-turn-helix domain-containing protein (54/69; 0.039; WP_001088243.1)	15931	15788	144	-	1
17	hypothetical protein	Ash family protein (100/68; 2e-10; WP_077776853.1)<br>Immunity region (100/66; 2e-09; EGW81362.1)	15986	16099	114	+	3
18	FIG00641704: hypothetical protein	Ash family protein (72/81; 2e-06; WP_089576887.1)	16517	16645	129	+	3
19	orf; Unknown function	[c]	16697	16870	174	+	4
20	orf; Unknown function	[c]	17172	17038	135	-	2
21	hypothetical protein	Transposase OrfB (99/100; 2e-07; KGM67713.1)<br>Integrase (71/81; 3e-04; KHE07974.1)	17251	17364	114	+	1
22	hypothetical protein	Colicin immunity protein (99/98; 1e-88; WP_077897121.1)<br>ShiD (99/98; 2e-88; AAD44741.1)<br>ColV-immunity protein (74/96; 4e-43; AIF92867.1)<br>Neuotensin receptor R8 (31/64; 8e-09; WP_087893235.1)<br>Flagellin biosynthesis protein FlgM (30/46; 0.055; WP_094248784.1)	17680	18078	399	+	4
23	hypothetical protein	[c]	18123	18251	129	+	2

24	hypothetical protein	Putative membrane protein (38/100; 0.043; EKJ22617.1)	18555	18406	150	-	3
25	FIG00642259: hypothetical protein	Putative ShiA-like protein (51/96; 8e-41; KDT77211.1)	18964	18548	417	-	3
26	ShiA homolog		19314	19147	168	-	3
27	hypothetical protein	c	19492	19376	117	-	2
28	ShiA homolog		19637	19494	144	-	3
29	hypothetical protein	DUF2251 domain-containing protein (60/73; 3e-06; WP_001563270.1)	20079	20228	150	+	2
30	TonB-dependent receptor		20418	22505	2088	+	4
31	Outer membrane vitamin B12 receptor BtuB		24695	22851	1845	-	3
32	hypothetical protein	Tansposase domain protein (100/100; 6e-34; EGW86064.1)	25193	25360	168	+	1
33	hypothetical protein	c	25392	25520	129	+	2
34	hypothetical protein	c	25853	25596	258	-	2
35	N-acetylgalactosamine 6-sulfate sulfatase (GALNS)		27040	25922	1119	-	3
36	N-acetylgalactosamine 6-sulfate sulfatase (GALNS)		27608	27156	453	-	3
37	Outer membrane porin OmpF		28839	27703	1137	-	3
38	hypothetical protein	c	28934	29104	171	+	2
39	hypothetical protein	c	29271	29429	159	+	2
40	hypothetical protein	Mobile element protein (70/86; 6e-11; KGI47791.1)<br>IS66 family element; Orf2 protein (70/86; 8e-11; EIH91436.1)<br>Putative transposase (93/53; 2e-09; KDY73070.1)<br>Effector protein (91/59, 4e-09; ETE13342.1)<br>ISpsy5; Orf1 (91/59; 4e-09; EFW61180.1)	29844	29632	213	-	1
41	hypothetical protein	Phage protein (68/47; 9e-10; WP_001559050.1)	30147	29902	246	-	2
42	hypothetical protein	c	30270	30413	144	+	2
43	hypothetical protein	c	30602	30456	147	-	2
44	hypothetical protein	c	30777	30959	183	+	2
45	Type III secretion transcriptional regulator HilD		30976	31713	738	+	3
46	hypothetical protein	c	31977	31795	183	-	2
47	hypothetical protein	c	32198	32001	198	-	2
48	hypothetical protein	c	32369	32593	225	+	2
49	Per-activated serine protease autotransporter enterotoxin EspC		32612	33184	573	+	3
50	orf, conserved hypothetical protein	c	675	250	426	-	4

51	FIG00639161: hypothetical protein	Aec60 (99/92; 1e-83; AAW51743.1)	1055	672	384	-	4
52	hypothetical protein	Transposase IS66 family protein (51/87; 2e-05; KDA77033.1)	1273	1139	135	-	1
53	Intergenic-region protein		1885	1316	570	-	4
54	FIG00639191: hypothetical protein	c	2089	2286	198	+	2
55	Haemolysin expression modulating protein		2772	2632	141	-	4
56	hypothetical protein	Low-affinity inorganic phophate transporter 2 (53/81; 2e-05; CTT80184.1) Transposases (61/80; 4e-05; AKP87350.1) Hemolysin activation protein (57/71; 9e-05; WP_074434862.1) Hemolysin expression modulator Hha (42/81; 0,005; WP_072021076.1)	2793	2939	147	+	3
57	hypothetical protein	Transcriptional regulator (65/96; 1e-07; OKT06225.1)	3392	3279	114	-	3
58	FIG00638000: hypothetical protein	AlpA family transcriptional regulator (98/100; 9e-63; WP_077778199.1) Helix-turn-helix domain-containing protein (98/96; 6e-60; WP_077879649.1)	3461	3739	279	+	4
59	FIG00638808: hypothetical protein	Malate transporter (99/99; 4e-72; WP_072097289.1) Aec62 (90/99; 1e-64; EGW80519.1)	3826	4140	315	+	4
60	Transposase InsN for insertion sequence element IS911		4183	4494	312	+	1
61	Transposase InsO for insertion sequence element IS911		4614	5360	747	+	1
62	FIG00638808: hypothetical protein	Aec62 (99/100; 4e-66; EGX14768.1)	5381	5689	309	+	4
63	hypothetical protein	Inovirus Gp2 family protein (43/67; 0.020; WP_074528747.1)	5968	5783	186	-	2
64	Uncharacterized protein YfjI		7495	6011	1485	-	3
65	hypothetical protein	c	7645	7887	243	+	2
66	FIG00640424: hypothetical protein	c	8049	7927	123	-	2
67	hypothetical protein	c	8474	8301	174	-	2
68	Uncharacterized protein YeeP		8725	9597	873	+	4
69	hypothetical protein	Diffuse adherence adhesion (85/100; 2e-25; EKJ54187.1) Antigen 43 (85/94; 2e-23; OSL31089.1)	9909	9745	165	-	3

		CP4-44 prophage; antigen 43 phase- variable biofilm formation autotransporter (85/94; 4e-22; BAJ43791.1) Putative autotransporter (29/100; 0.002; EGI40711.1)					
70	Antigen 43		9925	13044	3120	+	4
71	Inner membrane protein YeeR		13165	15681	2517	+	3
72	FIG00639908: hypothetical protein	[c]	15757	16212	456	+	2
73	FIG074102: hypothetical protein	Putative cytoplasmic protein (98/100; 9e-51; OSK21442.1) DUF905 domain-containing protein (98/100; 9e-51; WP_00119729.1)	16291	16524	234	+	3
74	UPF0380 proteins YafZ and homologs		16624	17442	819	+	4
75	Antirestriction protein klcA		17497	17982	486	+	4
76	UPF0758 protein YeeS		17983	18474	492	+	4
77	Uncharacterized protein YeeT		18537	18758	222	+	4
78	FIG00639142: hypothetical protein	[c]	18758	18871	114	+	2
79	YeeU protein (antitoxin to YeeV)		18921	19295	375	+	4
80	YeeV toxin protein		19342	19719	378	+	4
81	Z5092 protein		19716	20204	489	+	3
82	FIG00640476: hypothetical protein	DUF957 domain-containing protein (82/99; 5e-41; WP_012777794.1) Z1225 protein (82/96; 6e-40; CAD33791.1) Aec78 (82/94; 2e-37; AQZ79575.1)	20171	20413	243	+	4
83	Z1226 protein		20498	21340	843	+	4
84	hypothetical protein	[c]	21587	21468	120	-	2
	tRNA-Phe-GAA		21513	21534	22	+	tRNA

[a] CDS reading direction: + forward, - reverse

[b] assigned groups for predicted CDS function: 1) mobility, 2) hypothetical/ unknown, 3) metabolism/ fitness, 4) virulence factor

[c] no further classification of the hypothetical protein by blastx analysis possible

Table S5. Annotation and coding sequences (CDS), gene sequence length and direction of the *selC* tRNA gene associated part of contig 22 of strain 17584/1.

CDS	Function		Start	Stop	Length [bp]	Strand[a]	Group[b]
	RASTk annotation	Blastx analysis of hypothetical proteins (coverage/identity (%); E-value; Accession No.)					
	tRNA-SeC(p)-TCA		228	322	95	+	tRNA
1	Phage integrase		622	1806	1185	+	4
2	hypothetical protein	[c]	2105	2221	117	+	2
3	ShiA homolog		2218	3261	1044	+	4
4	hypothetical protein	[c]	3606	3484	123	-	2
5	Putative phosphoethanolamine transferase YjgX		4988	3612	1377	-	4
6	FIG00641722: hypothetical protein	DUF1705-domain (bacterial membrane protein) (86/100; 5e-13; WP_021565883.1) Phosphoethanolamin transferase (86/100; 2e-12; KZJ93053.1) metal-dependent hydrolase (86/100; 2e-12; WP_021524620.1)	4996	5130	135	+	3
7	Peptide transport periplasmic protein sapA (TC 3.A.1.5.5)		6035	5289	747	-	4
8	Integrase		6273	6443	171	+	1
9	FIG01045390: hypothetical protein	Restriction endonuclease (84/95; 1e-64; OAC36964.1)	6637	6990	354	+	3
10	hypothetical protein	EaeI restriction endonuclease (99/92; 0.0; AAB95338.1)	7632	8588	957	+	3
11	DNA-cytosine methyltransferase (EC 2.1.1.37)		9498	8569	930	-	3
12	hypothetical protein	[c]	9843	9715	129	-	3
13	Mobile element protein		10072	10326	255	+	1
14	Mobile element protein		10488	10814	327	+	4
15	Mobile element protein		10814	11293	480	+	4
16	hypothetical protein	[c]	11625	11506	120	-	4
17	Retron-type RNA-directed DNA polymerase (EC 2.7.7.49)		11885	13408	1524	+	4
18	Mobile element protein		13504	13977	474	+	4
19	hypothetical protein	[c]	14443	14060	384	-	2
20	FIG00613801: hypothetical protein	Hemagglutinin (99/99; 1e-98; KKO37879.1)	14884	14444	441	-	3
21	Putative member of ShlA/HecA/FhaA exoprotein family		15119	14943	177	-	3

22	FIG00763902: hypothetical protein	DUF2569 (93/57; 5e-64; WP_049294625.1)	15675	15154	522	-	2
23	FIG00613801: hypothetical protein	Filamentous hemagglutinin (86/91; 5e-27; WP_021572694.1)	15971	15690	282	-	3
24	hypothetical protein	c	16218	15976	243	-	2
25	Putative large exoprotein involved in heme utilization or adhesion of ShlA/HecA/FhaA family		16639	16205	435	-	3
26	hypothetical protein; Hypothetical gene	c	17588	17103	486	-	2
27	Putative large exoprotein involved in heme utilization or adhesion of ShlA/HecA/FhaA family		27364	17585	9780	-	3
28	Hemolysin activator protein precursor		29143	27377	1767	-	4
29	hypothetical protein	c	29186	29323	138	+	2
30	FIG00641476: hypothetical protein	c	29740	29507	234	-	4
31	FIG00641990: hypothetical protein	Putative DNA-binding protein (99/91; 2e-141; CBG36016.1)	30472	29837	636	-	4
32	FIG00641784 hypothetical protein	c	31154	30549	606	-	4
33	hypothetical protein	Putative membrane protein (97/100; 7e-18; KDU01312.1)	31296	31177	120	-	3
34	FIG00638000: hypothetical protein	Transcriptional regulator, AlpA family (98/100; 2e-43; EIH45477.1)<br>prophage CP4-57 regulatory protein family protein (98/100; 3e-43; EGW88643.1)	31528	31325	204	-	4
35	FIG00639802: hypothetical protein	c	32202	31630	573	-	4
36	hypothetical protein	c	32390	32262	129	-	2
37	FIG01048891: hypothetical protein	c	33014	32448	567	-	4
38	hypothetical protein	c	34501	35853	1353	+	2
39	Mobile element protein		36535	36092	444	-	1
40	hypothetical protein	c	36758	36618	141	-	2
41	Intergenic-region protein		37649	36918	732	-	4
42	hypothetical protein	c	38179	37991	189	-	2
43	hypothetical protein	DNA-binding protein (99/99; 3e-92; KFH93356.1)	38319	38849	531	+	3
44	hypothetical protein	c	38861	38989	129	+	2
45	hypothetical protein	c	39136	39252	117	+	2
46	hypothetical protein	c	39537	39659	123	+	2
47	FIG00639329: hypothetical protein	c	41188	39965	1224	-	4
48	NgrB		41289	42173	885	+	4
49	FIG00638087: hypothetical protein	c	42234	42347	114	+	2

50	FIG00641766: hypothetical protein	Transcriptional regulator (99/100; 1e-164; WP_001282919.1)	42376	43056	681	+	4
51	FIG00637880: hypothetical protein	[c]	43099	43881	783	+	4
52	UPF0401 protein YkfF	Phage protein (98/97; 3e-34; WP_001513976.1)	43887	44120	234	+	4
53	UPF0380 proteins YafZ and homologs	Phage protein (99/99; 0.0; WP_021530762.1)	44210	45028	819	+	4
54	Antirestriction protein klcA		45120	45605	486	+	4
55	UPF0758 protein YeeS		45620	46096	477	+	4
56	Uncharacterized protein YeeT		46159	46380	222	+	4
57	YeeU protein (antitoxin to YeeV)		46458	46823	366	+	4
58	YeeV toxin protein		46913	47290	378	+	4
59	FIG00640476: hypothetical protein	Phage protein (91/98; 2e-85; EQQ97275.1)	47287	47709	423	+	4
60	Z1226 protein	Restriction methylase (99/99; 0.0; WP_001280503.1)	47794	48636	843	+	4
	hypothetical protein		48972	48859	114	-	2
	Sugar efflux transporter B		49432	50616	1185	+	3

[a] CDS reading direction: + forward, - reverse

[b] assigned groups for predicted CDS function: 1) mobility, 2) hypothetical/ unknown, 3) metabolism/ fitness, 4) virulence factor

[c] no further classification of the hypothetical protein by blastx analysis possible

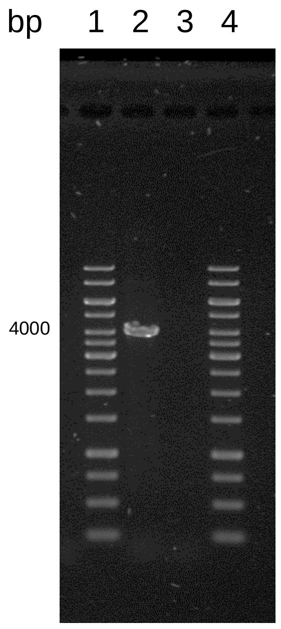

Figure S6: PCR detection of the Per-activated serine protease autotransporter enterotoxin EspC gene in strain 17584/1 (Lane 2, 3993 bp). Lane 1 and 4: GeneRuler 1 kb DNA ladder from Thermo Scientific; Lane 3: No template control. Primer: espC_Signal_for: 5’-GCACTCACTCTTTCCGGACAGATAATAC-3’; espC_rev: 5’-TCAGAACGAGTAACGGAAG TTAGCG-3’. The template genomic DNA was prepared using GenElute™ Bacterial Genomic DNA Kit (Sigma-Aldrich). The PCR was performed with Phusion High-Fidelity DNA Polymerase (Thermo Scientific) according to manufacturer recommendations.
